# Expression of expanded GGC repeats within *NOTCH2NLC* causes cardiac dysfunction in mouse models

**DOI:** 10.1186/s13578-023-01111-6

**Published:** 2023-08-29

**Authors:** Yongcheng Pan, Ying Jiang, Juan Wan, Zhengmao Hu, Hong Jiang, Lu Shen, Beisha Tang, Yun Tian, Qiong Liu

**Affiliations:** 1 Department of Neurology, Xiangya Hospital, Central South University, Changsha, 410008 Hunan China; 2 Key Laboratory of Hunan Province in Neurodegenerative Disorders, Central South University, Changsha, 410008 Hunan China; 3 Centre for Medical Genetics & Hunan Key Laboratory of Medical Genetics, School of Life Sciences, Central South University, Changsha, 410078 Hunan China; 4 Department of Neurology, Multi-Omics Research Center for Brain Disorders, The First Affiliated Hospital, Hengyang Medical School, University of South China, Hengyang, 421000 Hunan China; 5 National Clinical Research Center for Geriatric Disorders, Xiangya Hospital, Central South University, Changsha, 410008 Hunan China; 6 Department of Geriatrics, Xiangya Hospital, Central South University, Changsha, 410008 Hunan China

**Keywords:** *NOTCH2NLC* gene, GGC repeat expansion, NOTCH2NLC-polyG inclusions, Cardiomyocyte, Cardiac dysfunction, Mitochondria

## Abstract

**Background:**

Neuronal intranuclear inclusion disease (NIID) is a rare neurodegenerative disorder characterized by widespread intranuclear inclusions in the nervous system as well as multiple visceral organs. In 2019, expanded GGC repeats within the 5′ untranslated region of the *NOTCH2NLC* gene was identified as the causative factor. NIID is a heterogeneous disorder with variable clinical manifestations including cognitive impairment, cerebellar ataxia, parkinsonism, paroxysmal symptoms, autonomic dysfunction, and muscle weakness. Although NIID primarily affects the central and peripheral nervous systems, growing evidence suggests potential cardiac abnormalities in NIID. However, the link between expanded GGC repeats within *NOTCH2NLC* and cardiac dysfunction remains uncertain.

**Results:**

In this study, we utilized two transgenic mouse models, expressing *NOTCH2NLC-(GGC)*_*98*_ ubiquitously or specifically in cardiomyocytes, and identified p62 (also known as sequestosome 1, SQSTM1)-positive intranuclear NOTCH2NLC-polyG inclusions in cardiomyocytes in two mouse models. We observed that both models exhibited cardiac-related pathological and echocardiographic changes, albeit exhibiting varying degrees of severity. Transcriptomic analysis revealed shared downregulation of genes related to ion channels and mitochondria in both models, with the cardiomyocyte-specific mice showing a more pronounced downregulation of mitochondria and energy metabolism-related pathways. Further investigations revealed decreased expression of mitochondria-related genes and electron transport chain activity. At last, we conducted a retrospective review of cardiac-related examination results from NIID patients at our hospital and also identified some cardiac abnormalities in NIID patients.

**Conclusions:**

Our study provided the first in vivo evidence linking GGC repeat expansions within *NOTCH2NLC* to cardiac abnormalities and highlighted the contribution of mitochondrial dysfunction in the development of cardiac abnormalities.

**Supplementary Information:**

The online version contains supplementary material available at 10.1186/s13578-023-01111-6.

## Background

Neuronal intranuclear inclusion disease (NIID) is a rare neurodegenerative disorder characterized by widespread intranuclear inclusions in the nervous system as well as multiple visceral organs [1, 2]. The intranuclear inclusions are positive for p62, ubiquitin and ubiquitin-related protein [2–4]. In 2019, several studies independently identified expanded GGC repeats within the 5′ untranslated region (UTR) of the *NOTCH2NLC* gene as the causative factor associated with NIID [3–6]. Subsequent research had shown these expanded GGC repeats could encode toxic repeat proteins, such as NOTCH2NLC-polyglycine (polyG for short), disrupting normal cellular functions and causing toxicity in cellular and animal models, leading to NIID-like pathological manifestations and behavioral features [7–12].

NIID is a heterogeneous disorder with variable clinical manifestations including cognitive impairment, cerebellar ataxia, parkinsonism, paroxysmal symptoms, autonomic dysfunction, and muscle weakness [13–21]. Although NIID primarily affects the central and peripheral nervous systems, growing evidence suggests potential non-nervous system involvement, including the circulatory, respiratory, urinary, and digestive systems [22–24]. Of particular interest is the potential impact on the circulatory system in NIID.

Several reports have indicated the potential occurrence of various cardiac abnormalities in NIID patients, including cardiac insufficiency, valvular regurgitation, postural hypotension, and paroxysmal chest distress [22, 25]. The presence of cardiac abnormalities in NIID patients had been documented as early as 1991 [25]. Postmortem examinations of a NIID patient who died at the age of nine revealed intranuclear inclusions in cardiac myocytes. This patient also showed dilated heart, diffused fibrosis, and hypertrophy of myocytes, supporting the notion of cardiomyopathy associated with NIID [25]. In addition, recent studies have also reported cardiomyopathy in patients with oculopharyngodistal myopathy type 3 (OPDM 3) [26], another repeat expansion disorder involving GGC repeat expansions within *NOTCH2NLC* [18, 27]. These observations suggest the potential involvement of cardiac abnormalities within *NOTCH2NLC*-related repeat expansion disorders [23, 24]. However, a direct link between the cardiac abnormalities and the expanded GGC repeats within the 5′ UTR of the *NOTCH2NLC* gene is yet to be established.

In this study, we utilized two transgenic mouse models, expressing *NOTCH2NLC* with 98 GGC repeats, ubiquitously from the embryonic stage (EIIa;NOTCH2NLC-(GGC)_98_, EIIa-tg for short) or specifically in cardiomyocytes in adulthood (Myh6;NOTCH2NLC-(GGC)_98_, Myh6-tg for short) respectively, to investigate their impact on cardiomyocyte pathology and cardiac function. Both mouse models exhibited p62-positive intranuclear aggregates of PolyG in cardiomyocytes. EIIa-tg mice displayed pathological changes but retained preserved left ventricular systolic function. In contrast, Myh6-tg mice showed contractile dysfunction and chamber dilation. Transcriptomic sequencing of the hearts from both transgenic mouse models and age-matched control mice revealed the potential contribution of mitochondrial dysfunction and dysregulation of the metabolic pathway to cardiac functional abnormalities. Our retrospective analysis also identified cardiac abnormalities in NIID patients. This study provides the first evidence in the mouse model linking expanded GGC repeats within *NOTCH2NLC* to cardiac dysregulation.

## Results

### Expression of NOTCH2NLC-(GGC)_98_ produces polyG inclusions in the cardiomyocytes of EIIa-tg mice

**Fig. 1:**
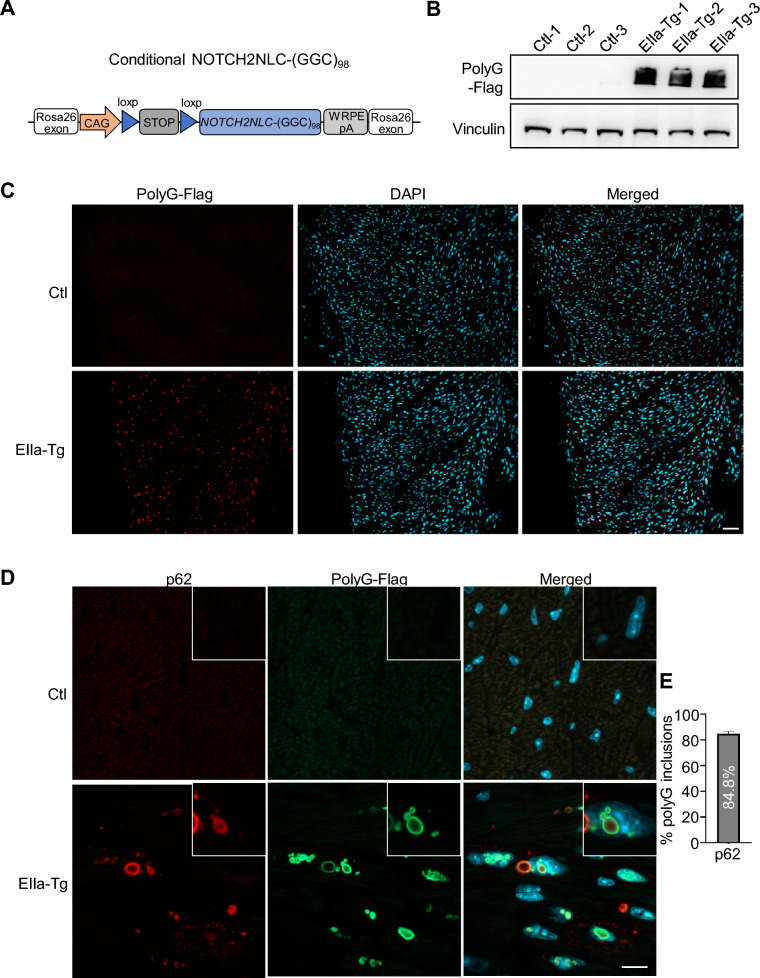
Expression of *NOTCH2NLC-(GGC)*_*98*_ produces polyG inclusions in the cardiomyocytes of EIIa-tg mice. **A** Schematic diagram of *NOTCH2NLC-(GGC)*_*98*_ construct. The conditional transgenic mice were obtained by inserting the loxP-stop-loxP-*NOTCH2NLC-(GGC)*_*9*8_ into the Rosa26 site. **B** Immunoblotting with an anti-Flag antibody in the hearts of control and EIIa-tg mice at P30. Vinculin was used as a loading control. **C** Immunofluorescent staining against Flag in the hearts of control and EIIa-tg mice at P30. Red, flag (polyG); cyan, DAPI. Scale bar, 50 μm. **D** Co-immunofluorescent staining against Flag and p62 in the hearts of control and EIIa-tg mice at P30. Red, p62; Green, flag (polyG); cyan, DAPI. Scale bar, 10 μm. **E** Colocalization of p62 with polyG inclusions in the heart of EIIa-tg mice. N = 3 mice per group, three images per mouse were used to count

Several reports have shown the presence of intranuclear inclusions within the cardiomyocytes of NIID patients [25] and cardiac issues have been reported in patients with expanded GGC repeats within *NOTCH2NLC* [22, 25, 26]. In our previous study, we established a conditional transgenic mouse model, in which the loxP-stop-loxP-*NOTCH2NLC-(GGC)*_*9*8_ was inserted into the Rosa26 site (Fig. 1A) [11]. The conditional transgenic mice were then crossed with EIIa-Cre mice to express *NOTCH2NLC-(GGC)*_*9*8_ ubiquitously (EIIa-tg) [11]. Intriguingly, the EIIa-tg mice exhibited increased chest movements and rapid breathing in the later stages of disease progression (Additional file 1: Video S1), possibly indicating cardiopulmonary dysfunction. This led us to hypothesize that expanded GGC repeats within *NOTCH2NLC* may lead to intranuclear inclusions and cause damage to cardiomyocytes in the EIIa-tg mouse model.

To test this hypothesis, we first assessed the expression of mutant polyG in the hearts of EIIa-tg mice. Western blot using an anti-flag antibody (the flag-tag was fused to PolyG), whose efficiency and specificity had been previously demonstrated [11], revealed the presence of aggregated mutant polyG remained in the stacking gel in EIIa-tg mice at postnatal days 30 (P30), but not in control mice (conditional NOTCH2NLC-(GGC)_98_ mice without EIIa-cre expression, Fig. 1B). The soluble mutant polyG proteins could not be detected and the data were not shown here. Additionally, we performed immunofluorescent staining to confirm the expression of mutant polyG in the heart. Positive staining was observed to be widely distributed in cardiomyocytes (Fig. 1C). At higher magnification, micrographs showed that mutant polyG formed large intranuclear and perinuclear inclusions (Fig. 1D) as seen in the nervous system [11]. Consistently, co-staining with anti-p62 revealed that most of the polyG inclusions (Fig. 1E, about 84.8%) were p62 positive, reminiscent of what has been observed in NIID patients [2, 4].

### Impaired ventricular structure and preserved LV systolic function in the EIIa-tg mice

**Fig. 2:**
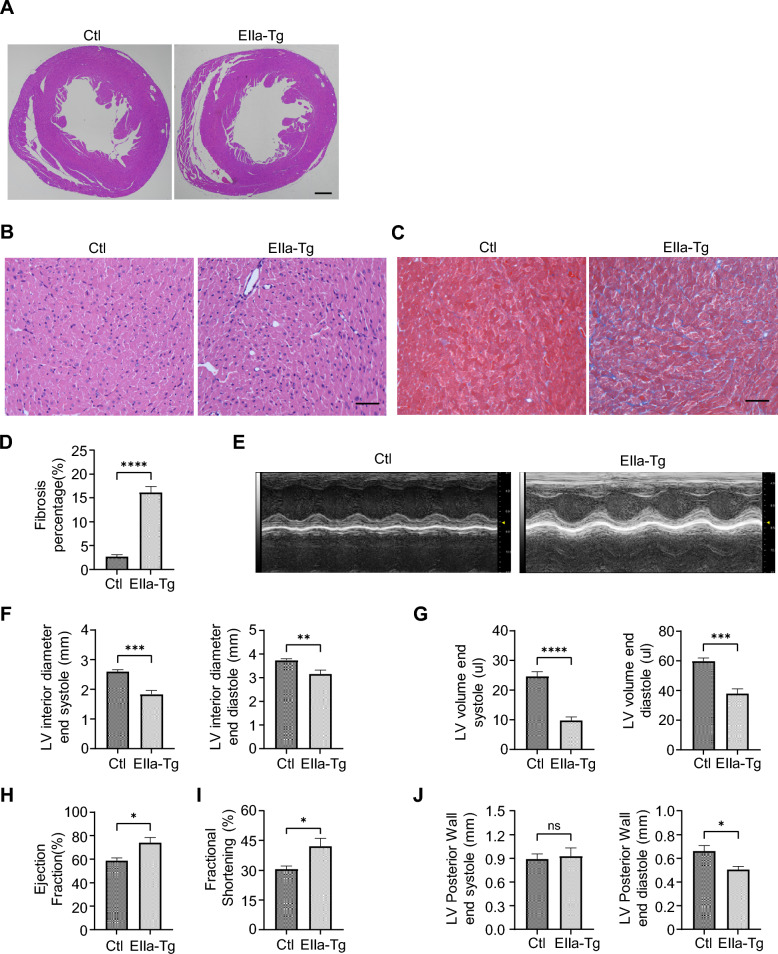
PolyG proteins impair ventricular structure in the EIIa-tg mice. **A** Representative 2-chamber cross-section, Hematoxylin-eosin stained at P30. Scale bar, 500 μm.** B** Hematoxylin–eosin staining showed mild infiltrating cells in the transgenic mice at P30. Scale bar, 40 μm. **C** Representative images from Masson-stained transgenic mice and control mice at P30. Scale bar, 40 μm. **D** Quantification of myocardial fibrotic area. **P < 0.01. **E** Representative M-mode echocardiography of the LV chamber. **F**–**J** Assessment of LVID at end systole and diastole (**F**), LVV at end systole and diastole (**G**), EF (%) (**H**), FS (%) (**I**), and LVPW thickness at end systole and diastole (**J**). n = 6 mice per group for all experiments. Data are presented as mean ± SEM. *P < 0.05, **P < 0.01, ***P < 0.001, ****P < 0.0001, Student’s t test. *LV* left ventricular, *LVID* left ventricular internal dimension, *LVV* left ventricular volume, *EF* ejection fraction, *FS* fractional shortening, *LVPW* left ventricular posterior wall

In our previous report, we demonstrated that the expression of *NOTCH2NLC-(GGC)*_*98*_ could lead to gastrocnemius muscle degeneration and impaired muscle function in the EIIa-tg mice [11]. Given these findings, we aimed to investigate whether the presence of mutant polyG proteins would also influence cardiac function. Histological analysis of EIIa-tg mice revealed mild infiltrating cells in the transgenic heart sections (Fig. 2A, B). Then we performed Masson staining and found an increased positive area of cardiac interstitial fibrosis (Fig. 2C, D). To evaluate cardiac function longitudinally, we conducted echocardiography. Left ventricular (LV) internal dimension at end-diastole and end-systole (LVID; d and LVID; s) were substantially decreased in EIIa-tg mice compared to control mice (Fig. 2E, F). Although these transgenic mice displayed decreased LV volume at end-diastole and end-systole (LVV; d and LVV; s), the LV ejection fraction (EF) and LV fractional shortening (FS) were significantly increased (Fig. 2G, H), indicating ventricular systolic compensation. In addition, LV posterior wall thickness at end-diastole (LVPW; D) was decreased, while LV posterior wall thickness at end-systole (LVPW; S) was comparable to that of control mice (Fig. 2I). Taken together, these results indicated that mutant polyG proteins caused pathologic cardiac remodeling in the EIIa-tg mice.

### Cardiomyocyte-specific expression of NOTCH2NLC-(GGC)_98_ produces polyG inclusions in the cardiomyocytes

**Fig. 3:**
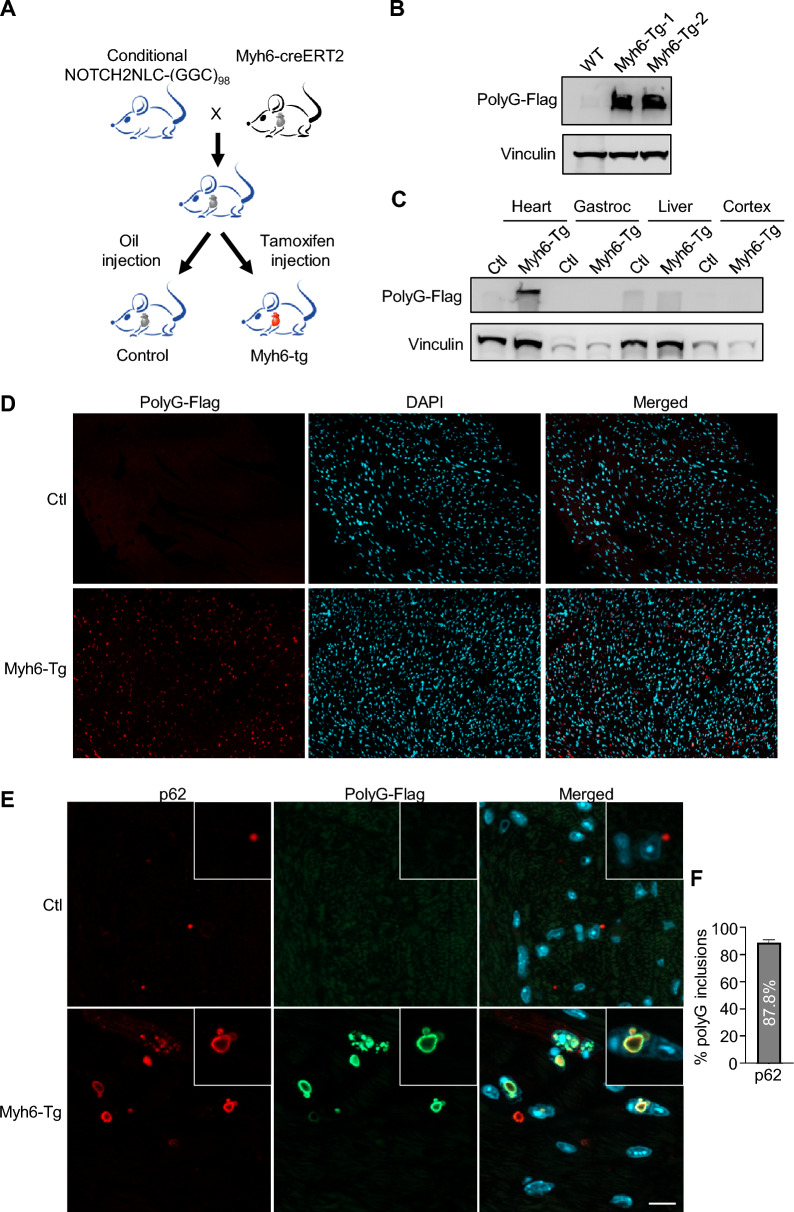
Generation of Myh6-tg mice that express mutant polyG in cardiomyocytes.** A** Schematic diagram of generation of the Myh6-tg mice**. B** Representative immunoblot for polyG in the heart of wild-type and Myh6-tg. Vinculin was used as a loading control. **C** Immunoblotting analysis of polyG in the oil or tamoxifen injected heart, gastrocnemius, liver, and cortex. Vinculin was used as a loading control.** D** Immunofluorescent staining against Flag in the hearts of control and Myh6-tg mice at P90. Red, flag (polyG); cyan, DAPI. Scale bar, 50 μm. **E** Co-immunofluorescent staining against Flag and p62 in the hearts of control and Myh6-tg mice at P90. Red, p62; Green, flag (polyG); cyan, DAPI. Scale bar, 10 μm. **F** Colocalization of p62 with polyG inclusions in the heart of Myh6-tg mice. N = 3 mice per group, three images per mouse were used to count

To further validate the effects of *NOTCH2NLC-(GGC)*_*9*8_ expression on cardiomyocytes and cardiac function, we generated Myh6-tg mice by crossing conditional *NOTCH2NLC-(GGC)*_98_ mice with Myh6-creERT2 mice, which express a tamoxifen-inducible Cre recombinase under the promotor of alpha-myosin heavy chain promoter (αMHC or Myh6) in cardiomyocytes [28]. At postnatal day 60 (P60), Myh6-tg mice were injected with tamoxifen to induce selective expression of *NOTCH2NLC-(GGC)*_98_ in cardiomyocytes (Fig. 3A), while littermates were injected with corn oil as controls.

To verify the expression of mutant polyG, we performed western blot analysis using heart extracts from the Myh6-Tg and Control mice at P90 (30 days after injection). As expected, immunoblotting with an anti-Flag antibody showed the presence of aggregated mutant polyG proteins in the stacking gel of the Myh6-Tg mice, while no such signals were observed in the control mice (Fig. 3B). To assess the specificity of polyG expression driven by Myh6-cre, we examined the expression of polyG in other tissues, including gastrocnemius, liver, and cortex. The results showed that polyG was exclusively expressed in cardiomyocytes (Fig. 3C). Immunostaining further demonstrated the widespread distribution of polyG inclusions in the cardiomyocytes of the Myh6-ctg mice, whereas no positive signals were detected in the control mice (Fig. 3D). Furthermore, most of polyG inclusions (about 87.8%) were p62 positive (Fig. 3E, F), consistent with the observations in the EIIa-tg mice.

### Impaired ventricular structure and cardiac function in the Myh6-tg mice

**Fig. 4:**
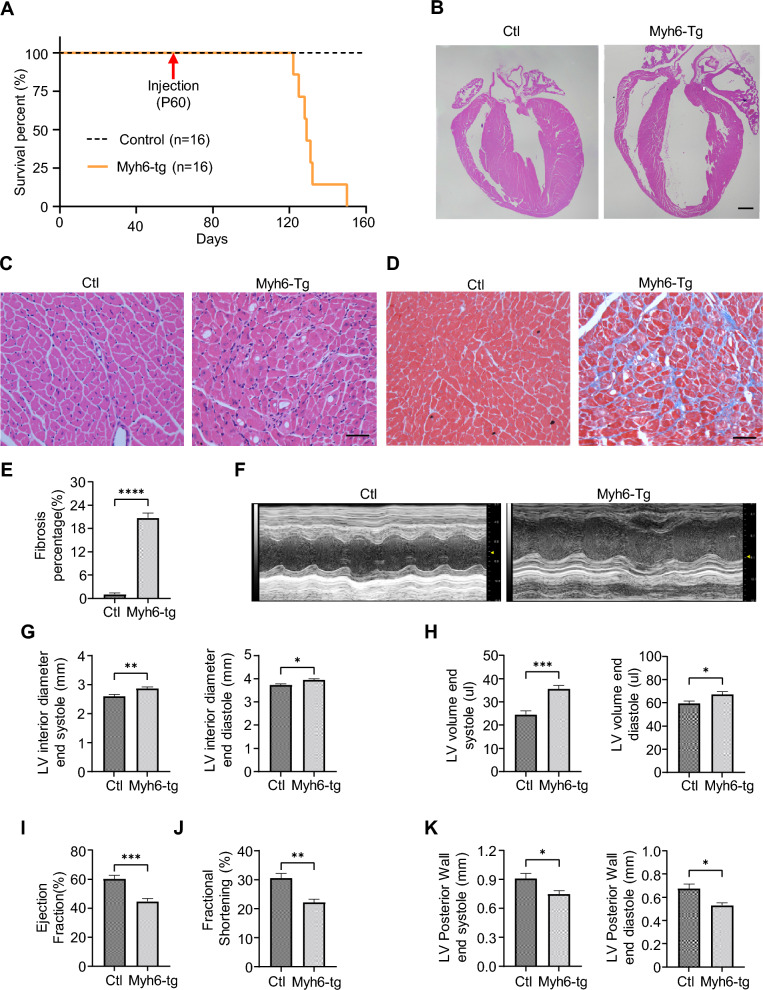
Mutant polyG proteins impair ventricular structure and function in the Myh6-tg mice_***.***_** A** Survival curve of Myh6-tg mice after tamoxifen or oil injection.** B** Representative 4-chamber cross-section, Hematoxylin-eosin stained at P90. Scale bar, 500 μm.** C** Hematoxylin-eosin staining showed infiltrating cells and vacuoles in the transgenic mice at P90. Scale bar, 40 μm. **D** Representative images from Masson-stained transgenic mice and control mice at P90. Scale bar, 40 μm. **E** Quantification of myocardial fibrotic area. **P < 0.01. **F** Representative M-mode echocardiography of the LV chamber. **G-K** Assessment of LVID at end systole and diastole (**G**), LVV at end systole and diastole (**H**), EF (%) (**I**), FS (%) (**J**) and LVPW thickness at end systole and diastole (**K**). N = 6 mice per group for all experiments. Data are presented as mean SEM. *P < 0.05, **P < 0.01, ***P < 0.001, using Student’s t test. *LV* left ventricular, *LVID* left ventricular internal dimension, *LVV* left ventricular volume, *EF* ejection fraction, *FS* fractional shortening, *LVPW* left ventricular posterior wall

The Myh6-tg mice showed no overt anomalies in appearance after tamoxifen injection. However, the mice died gradually after P120 (60 days after injection), regardless of gender (Fig. 4A). HE staining showed cardiac dilation and thinner interventricular septum and ventricular wall in the Myh6-tg mice at P90 compared to the control mice (Fig. 4B). Notably, the Myh6-tg mice displayed infiltrating inflammatory cells and vacuoles, indicating cardiomyocyte degeneration (Fig. 4C). Masson staining showed increased fibrosis and interstitial space in the heart, suggestive of ventricular structural remodeling (Fig. 4D, E).

Echocardiography was performed to assess heart function. At P90, the Myh6-tg mice showed significant LV chamber dilation, as evidenced by increased LVID; d and LVID; s, compared to the control mice (Fig. 4F, G). Additionally, LVV; d, and LVV; s were increased significantly (Fig. 4H). Consistent with the increased pathological LV remodeling, the cardiac contractile function was notably reduced, as indicated by decreased LVEF and LVFS in the Myh6-tg mice (Fig. 4I). Furthermore, LVPW; D and LVPW; S were decreased compared to the control mice (Fig. 4J). These findings collectively demonstrate significant cardiac contractile dysfunction and chamber dilation when mutant polyG was specifically expressed in cardiomyocytes in adult mice.

### Transcriptomic profiling reveals shared and distinct molecular profiles related to cardiac dysfunction in the EIIa-tg and Myh6-tg mice

To explore the underlying mechanisms leading to cardiac dysfunction upon the expression of *NOTCH2NLC-(GGC)*_*98*_ in cardiomyocytes, we performed RNA-seq analysis on heart tissues. Notably, we observed both shared and distinct pathological, structural, and functional changes between the EIIa-tg and Myh6-tg mouse models. To comprehensively investigate these differences, we conducted RNA-seq analysis on heart tissues obtained from EIIa-tg mice at P30 and Myh6-tg mice at P90 (30 days after the first tamoxifen injection), along with age-matched control mice.

**Fig. 5:**
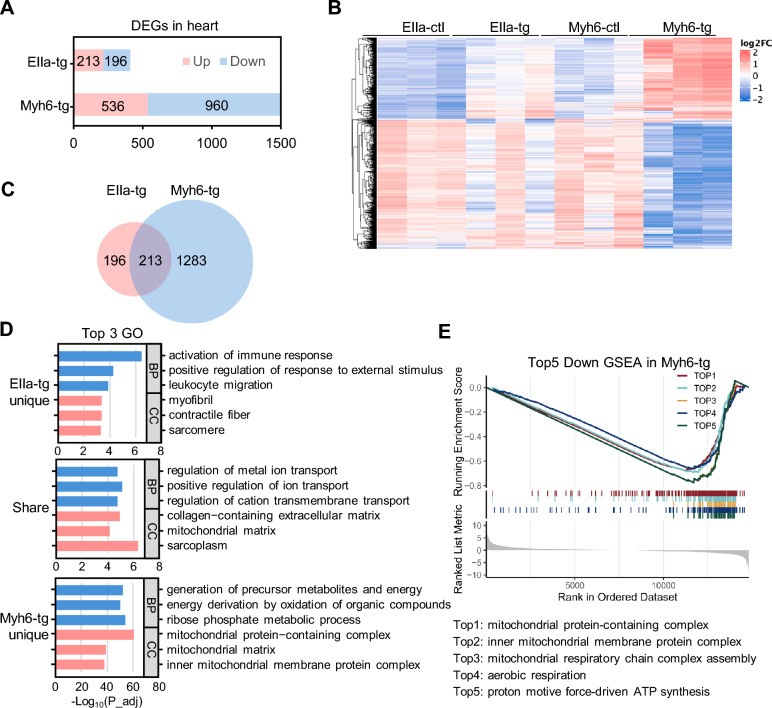
Transcriptomic profiling reveals shared and distinct molecular profiles related to cardiac dysfunction in EIIa-tg and Myh6-tg mice.** A** The numbers of down-regulated and up-regulated differentially expressed genes (DEGs) in the heart from EIIa-tg and Myh6-tg mice**.** Genes with adjusted *P* value (*P*_adj) < 0.01 and log_2_|FoldChange|≥ 1 were assigned as DEGs. **B** Heatmap of DEGs among all groups (n = 3 mice per group). **C** The overlapping of DEGs (both down-regulated and up-regulated) between the EIIa-tg and Myh6-tg mice. **D** Top 3 GO terms for DEGs exclusively identified in EIIa-tg, DEGs shared in both EIIa-tg and Myh6-tg mice, and DEGs exclusively identified in Myh6-tg. **E** Top 5 down-regulated GSEA pathways in Myh6-tg mice

The RNA-seq analysis revealed 409 significantly differentially expressed genes (DEGs) in the heart tissues of EIIa-tg mice and 1496 DEGs in the heart tissues of Myh6-tg mice when compared to their respective control groups (Additional file 2: Table S1). The significant increase in DEGs in Myh6-tg mice, over three-fold compared to EIIa-tg mice, indicated that Myh6-tg mice may experience more profound molecular alterations (Fig. 5A). The heatmap analysis further confirmed that the molecular profile of Myh6-tg mice is significantly distinct from the other groups (Fig. 5B). Among the 409 DEGs identified in EIIa-tg heart tissues, 213 genes were up-regulated, and 196 genes were down-regulated. These DEGs were found to be enriched in the regulation of immune response pathways based on Gene Ontology (GO) and Kyoto Encyclopedia of Genes and Genomes (KEGG) analysis (Additional file 3: Fig S1A), suggesting potential activation of immune responses in EIIa-tg mice. On the other hand, in Myh6-tg mice, we identified 960 down-regulated DEGs and 536 up-regulated DEGs among the 1496 DEGs detected. Notably, these DEGs were enriched in mitochondrion-related pathways and energy metabolism based on GO analysis and were associated with cardiomyopathy in KEGG analysis (Additional file 3: Fig S1B). These findings indicate that the expression of *NOTCH2NLC-(GGC)*_*98*_ in Myh6-tg mice may disrupt mitochondrial functions and energy metabolism in the heart, potentially contributing to cardiomyopathy development.

Taking into account our previous findings which revealed cardiac-related pathological and echocardiographic modifications in both EIIa-tg and Myh6-tg models, we conducted a comparative analysis of the DEGs (both down-regulated and up-regulated) between the two mouse lines (Fig. 5C). The gene sets unique to EIIa-tg mice was significantly enriched in the regulation of immune responses (Fig. 5D and Additional file 3: Fig S1C), indicating that immune response pathways. The overlapping gene sets between EIIa-tg and Myh6-tg mice was significantly enriched in ion transportation, and extracellular and mitochondrial matrix (Fig. 5D and Additional file 3: Fig S1D), which may suggest that the disturbance of intracellular and extracellular ion transportation may be relevant to the development of cardiac abnormalities in EIIa-tg and Myh6-tg mice. While the Myh6-tg-specific gene sets were notably enriched in mitochondrion-related pathways and energy metabolism (Fig. 5D and Additional file 3: Fig S1E). Furthermore, Gene Set Enrichment Analysis (GSEA) identified the most significantly down-regulated pathways in Myh6-tg mice were enriched in mitochondrial protein complexes, assembly of the mitochondrial respiratory chain, aerobic respiration, and proton motive force-driven ATP synthesis (Fig. 5E). These enrichments highlight the pronounced impact of disturbances in mitochondrial respiration and energy metabolism in the advanced stage of cardiac abnormalities observed in Myh6-tg mice.

Overall, our transcriptomic analysis provides valuable insights into the molecular mechanisms underlying cardiac dysfunction in both EIIa-tg and Myh6-tg mouse models. The shared changes in ion transportation and mitochondria suggest common underlying pathological pathways, while the differences in immune response regulation and mitochondrial function indicate distinct molecular signatures at different stages of the disease.

### Expression of *NOTCH2NLC-(GGC)*_*98*_ in cardiomyocytes disturbs mitochondrial respiration and energy metabolism in the heart

**Fig. 6:**
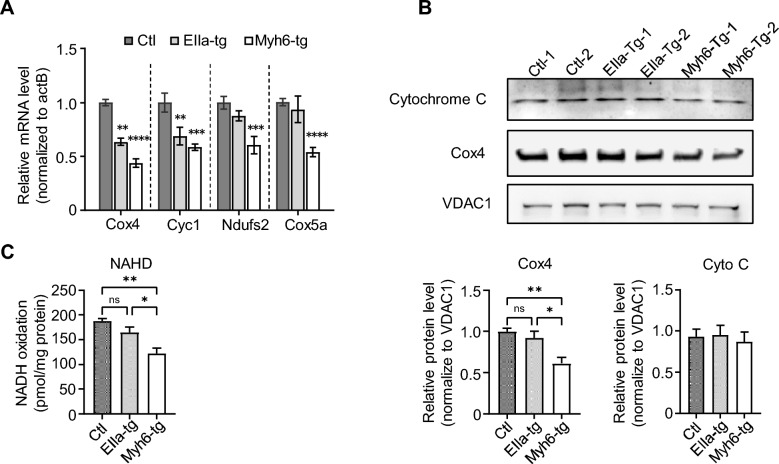
Expression of *NOTCH2NLC-(GGC)*_*98*_ in cardiomyocytes disturbs mitochondrial respiration and energy metabolism in the heart.** A** QPCR validation of RNA seq-identified DEG genes in EIIa-tg and Myh6-tg and control mice. N = 3 mice per group. *P < 0.05, ****P < 0.0001 by one-way ANOVA followed by Tukey's multiple comparisons test. **B** Immunoblot of COX4 and Cytochrome C (Cyto C) proteins in isolated ventricular mitochondria from EIIa-tg and Myh6-tg and control mice. VDAC1 was used as a loading control. Data are presented as mean ± SEM. N = 4 per group. *P < 0.05, **P < 0.005 by one-way ANOVA followed with Tukey's multiple comparisons test.** C** NADH oxidase activity was measured by the NADH consumption rate of isolated ventricular mitochondria from EIIa-tg and Myh6-tg and control mice. N = 4 per group. *P < 0.05, **P < 0.005 by one-way ANOVA followed with Tukey's multiple comparisons test

To further elucidate the impact of *NOTCH2NLC-(GGC)*_*98*_ on cardiomyocyte mitochondria, we selected some genes highly related to mitochondria [29] from the DEGs for validation through quantitative PCR (qPCR). The qPCR results showed that the mRNA level of *Cox4* and *Cyc1* was decreased in both EIIa-tg and Myh6-tg mice, while the mRNA level of *Ndufs2* and *Cox5a* was decreased only in Myh6-tg mice (Fig. 6A). To further corroborate these findings, we proceeded to investigate the expression levels of specific mitochondria-related proteins. Interestingly, western blot analysis showed a significant reduction in Cox4 protein expression, key enzymes of the electron transport chain within mitochondria, specifically in the hearts of Myh6-tg mice (Fig. 6B), indicating a critical role of the polyG protein in impacting the proper functioning of the mitochondrial respiratory chain. To provide functional validation of these observed molecular changes, we employed assay kits to quantitate the activity of NADH, an essential electron carrier involved in oxidative phosphorylation, within the mitochondrial complex. Remarkably, the NADH activity was found to be significantly decreased in the hearts of Myh6-tg mice (Fig. 6C), further supporting the notion that the polyG protein negatively affects mitochondrial metabolism.

Taken together, our findings suggest that the expression of *NOTCH2NLC-(GGC)*_*98*_ and the subsequent accumulation of polyG protein in cardiomyocytes lead to impaired mitochondrial function. The downregulation of mitochondria-related genes and reduced NADH activity collectively indicate a disruption in mitochondrial respiratory and energy metabolism in the hearts of Myh6-tg mice. These findings shed light on the molecular mechanisms underlying cardiac dysfunction induced by expanded GGC repeats in the *NOTCH2NLC* gene.

### Retrospective analysis suggests cardiac abnormalities in NIID patients

Considering the evidence from our mouse study that demonstrates the association of the expanded GGC repeats within *NOTCH2NLC* with cardiac dysfunction, we conducted a retrospective review of cardiac-related examination results from NIID patients at our hospital [13]. Our observations revealed a higher proportion of these patients presenting with cardiac abnormalities including biochemical, electrocardiographic, and echocardiographic abnormalities. Among 51 patients with NIID, 34 (66.7%) showed some form of cardiac abnormality (Table 1). Specifically, 6 patients (11.8%) had elevated creatine kinase levels and 5 patients (9.8%) had elevated creatine kinase-MB levels However, no myogenic changes were observed in the electromyogram results, indicating the possibility of myocardial damage in these patients. Moreover, 33 patients (64.7%) displayed electrocardiographic abnormalities, including 9 with cardiac arrhythmias and 28 with ischemic ST-T or T wave changes.

**Table 1:** Cardiac findings of NIID patients

	Total (n = 51)
Sex ratio (male/ female)	23/28
Age at onset, years, median (IQR)	57 (50–64)
Age, years, median (IQR)	65 (58–67)
Disease duration, years, median (IQR)	6 (2–11)
Creatine kinase, median (IQR), U/L	68.0 (44.5–117.1)
Creatine kinase- MB, median (IQR), U/L	10.9 (9.2–14.5)
Creatine kinase level abnormality	6/51 (11.8%)
Creatine kinase- MB level abnormality	5/51 (9.8%)
Electrocardiographic conduction abnormality	33/51 (64.7%)
Any cardiac abnormality	34/51 (66.7%)

There were seven NIID patients had received echocardiography examinations, and all of them presented with cardiac abnormalities (Additional file 3: Table S2). Two patients showed interventricular septal thickening, and six patients had mild mitral valve regurgitation, while all patients had tricuspid regurgitation, with one of them having severe tricuspid regurgitation. Regarding ventricular function, no patients showed ventricular systolic dysfunction, but a significant proportion (5/7, 71.4%) had diastolic dysfunction with an E/A ratio smaller than 1. Notably, three of the five patients with diastolic dysfunction had no history of hypertension or heart disease, suggesting that other factors might contribute to the diastolic dysfunction. In summary, the above findings suggest cardiac abnormalities in NIID.

## Discussion

In this study, we present the first and compelling evidence supporting the strong correlation between expanded GGC repeats in the *NOTCH2NLC* and cardiac dysfunction in mice. Firstly, in both EIIa-tg and Myh6-tg mouse models, expressing *NOTCH2NLC-(GGC)*_*98*_ ubiquitously or specifically in cardiomyocytes respectively, we observed the presence of abundant intranuclear and perinuclear polyG inclusions within cardiomyocytes. Notably, these polyG inclusions were found to colocalize with p62, a typical pathological marker in NIID. Secondly, histological examination of both mouse models revealed cardiac pathological abnormalities including cellular infiltration and fibrosis. Thirdly, both mouse models showed changed ventricular structure and cardiac function. Echocardiography found decreased LVID, LVV, and LVPW in EIIa-tg mice, while found increased LVID and LVV but decreased LVPW in Myh6-tg mice. Moreover, the significantly reduced LVEF and LVFS in the Myh6-tg mice indicated a severe impairment in cardiac contractile function. Collectively, the findings from both transgenic mouse models support our hypothesis that expanded GGC repeats in the *NOTCH2NLC* lead to cardiac dysfunction in mice.

Intriguingly, although both transgenic mouse models exhibited pathological changes and cardiac functional abnormalities mentioned above, they displayed distinct features. EIIa transgenic mice showed decreased LVID, LVV, and LVPW without obvious dilation or hypertrophy, while the Myh6-tg mice exhibited cardiac dilation with thinner interventricular septum and ventricular walls. Furthermore, the EIIa mice demonstrated elevated EF and FS, indicating a compensatory phase in cardiac function, whereas the Myh6-tg mice exhibited decreased EF and FS. The observed differences may be attributed to various factors. Firstly, the commencement of *NOTCH2NLC-(GGC)*_*98*_ expression diverges between the E2a-tg and Myh6-tg mice, with onset during the embryonic stage in the former and at P60 in the latter. Secondly, the expression of *NOTCH2NLC-(GGC)*_*98*_ is driven by EIIa-cre recombinase in the E2a-tg mice, while it is driven by tamoxifen-induced Myh6-Cre recombinase in the Myh6-tg mice, potentially influencing NOTCH2NLC-polyG levels. Thirdly, experimental observations were conducted at different time points, with E2a-tg mice examined at P30 (30 days after birth) and Myh6-tg mice at P90 (30 days after tamoxifen injection).

Transcriptomic analysis of these two mouse models has provided valuable insights into underlying mechanisms. GO and KEGG analysis suggested that the DEGs unique in EIIa-tg were enriched in the regulation of immune responses, while the overlapping DEGs were significantly enriched in ion transportation and extracellular and mitochondrial matrix. Notably, the DEGs specific to Myh6-tg mice were significantly enriched in mitochondrion-related pathways and energy metabolism. Furthermore, GSEA highlighted the down-regulation of crucial mitochondrial pathways in Myh6-tg mice, including the assembly of mitochondrial protein complexes, mitochondrial respiratory chain, aerobic respiration, and proton motive force-driven ATP synthesis. Consistently, western blot analysis and NADH oxidation activity assays confirmed reduced COX4 protein levels and impaired mitochondrial function in the hearts of Myh6-tg mice. Our findings strongly indicate the involvement of mitochondrial dysfunction in the development of cardiac dysfunction in these mouse models.

Although we were unable to directly investigate heart tissue from patients with *NOTCH2NLC*-related repeat expansion disorders due to limited access, previous studies have reported mitochondrial dysfunction in the skeletal muscle of patients with NIID, characterized by mitochondrial swelling and down-regulation of complex I-encoding genes [9]. Considering the high energy demands of both skeletal and cardiac muscles, and the presence of dysfunction in both tissues in our EIIa-tg mice, our findings suggest a potential link between *NOTCH2NLC*-related repeat expansion and mitochondrial dysfunction in cardiac muscle, which may contribute to the development of cardiac dysfunction. Further work will be needed to dissect the mechanism of how GGC repeat expansions within *NOTCH2NLC* lead to the down-regulation of mitochondria-related genes and disturbance in mitochondrial respiration and energy metabolism. Additionally, the potential interactions between the cardiac and neural systems in the context of *NOTCH2NLC*-related repeat expansion disorders merit investigation [30].

In a recent report, a patient diagnosed with *NOTCH2NLC*-related OPDM3, a neuromuscular disease, exhibited severe cardiac manifestations, including left ventricular systolic dysfunction with decreased left ventricular ejection fraction and elevated N-terminal pro-B type natriuretic peptide levels [26]. In our observation of NIID patients, most of whom did not show significant myogenic changes [13], we found a higher proportion of patients with various mild cardiac abnormalities, such as elevated creatine kinase or creatine kinase-MB levels, ischemic ST-T or T wave changes. These observations suggest that patients with muscle dagame may be more susceptible to cardiac involvement [31], consistent with the observations in our mouse model. Additionally, it is important to note that the degree and manifestation of cardiac involvement in NIID can vary widely among individuals and do not be present in all cases. This phenomenon is also observed in other repeat expansion disorders, such as myotonic dystrophy and Friedreich’s ataxia, which also exhibit cardiac abnormalities [32–35]. Various factors, such as repeat lengths, disease progression stage, and other genetic factors, could contribute to this variation [35–37]. Therefore, future research and clinical practice should pay more attention to monitoring the heart health of patients with *NOTCH2NLC*-related repeat expansion disorders.

## Conclusions

In summary, our study provided the first in vivo evidence linking GGC repeat expansions within *NOTCH2NLC* to cardiac abnormalities and highlighted the contribution of mitochondrial dysfunction in the development of cardiac abnormalities.

## Materials and methods

### Cardiac examinations on NIID patients

Clinical data were retrospectively collected from 51 NIID patients from June 2019 to June 2023 at Xiangya Hospital, Central South University. All participants underwent electrocardiographic examinations and myocardial enzyme detections. Seven of them received echocardiography examinations. Data were evaluated in comparison to reference values of a normative study in our laboratory. This study was approved by the Ethics Committee of the Xiangya Hospital (No. 202202050549). Written informed consent was obtained from all participants.

### Animals

The conditional transgenic *NOTCH2NLC-(GGC)*_*9*8_ mice were generated previously [11], in which the loxP-stop-loxP-*NOTCH2NLC-(GGC)*_*9*8_ was inserted into the Rosa26 site. To express *NOTCH2NLC-(GGC)*_*9*8_ ubiquitously or specifically in cardiomyocytes, the conditional transgenic mice were crossed with EIIa-Cre mice or Myh6-creERT2 mice. The EIIa-cre mice were used in our previous study [11] and the Myh6-creERT2 mice [28] were obtained from the Jackson Laboratory (JAX: 005657). The adult Myh6-tg mice were administered tamoxifen (20 mg/ml, dissolved in corn oil) or corn oil via intraperitoneal injection (injection dose: 75 mg/kg body weight) once every other day for a total of 3 injections. All mice were bred and maintained in the animal facility under specific pathogen-free conditions following the institutional guidelines of the Animal Care and Use Committee at Central South University.

### Antibodies

Primary antibodies used in this study include the following: Flag (Sigma-Aldrich, F1804), Flag (Cell Signaling Technology, 14793S), p62 (Abcam, AB56416), vinculin (Sigma-Aldrich, V9131), Cytochrome C (Cell Signaling Technology, 4272), COX4 (Cell Signaling Technology, 4844S), VDAC (Cell Signaling Technology, 4661). Secondary antibodies were donkey anti-rabbit and donkey anti-mouse Alexa Fluor 488 or 594 from Jackson ImmunoResearch.

### Western blot analysis

Mouse heart tissues were lysed in ice-cold RIPA buffer (50 mM Tris, pH 8.0, 150 mM NaCl, 1 mM EDTA pH 8.0, 1 mM EGTA pH 8.0, 0.1% SDS, 0.5% DOC, and 1% Triton X-100) containing protease inhibitor cocktail and PMSF. The lysates were sonicated and subjected to Western blot analysis with appropriate primary antibodies.

### Immunofluorescent staining

Mice were anesthetized and perfused intracardially with 0.9% NaCl, followed by 4% paraformaldehyde in 0.1 M phosphate buffer at pH 7.2. Isolated mouse brains were dehydrated in 30% sucrose and then sectioned at 30 μm. Brain sections were permeabilized with 0.3% Triton X-100/PBS at room temperature for 1 h and then applied to antigen retrieval with citric acid buffer (10 mM citric acid, 0.05% Tween 20, PH6.0) at 98 ℃ water bath for 15 min. Brain sections were blocked in 3% BSA in 0.3% Triton X-100/PBS for 1 h followed by incubation with primary antibodies at 4 ℃ overnight. After washing with 1X PBS, the sections were incubated with Alexa fluor-conjugated secondary antibodies and DAPI. Fluorescent images were captured with a Zeiss microscope.

### Hematoxylin-eosin and masson staining

For hematoxylin-eosin staining, paraffin-embedded heart sections (5 μm) were hematoxylin stained for 5 min and alcoholic fractionated with 1% hydrochloric acid for a few seconds, followed by eosin staining for 2 min. For Masson staining, heart sections were stained in Weiger iron hematoxylin solution for 8 min and graded with 0.5% hydrochloric acid for 15 s. Then the sections were stained with lychee red acid magenta solution for 8 min. After being treated with 1% phosphomolybdic acid for 5 min, the sections were stained with aniline blue solution for a further 5 min and followed by treated with 1% glacial acetic acid for 1 min.

### Echocardiography

Echocardiography was conducted with minor medications, following the protocol described in a previous study [38]. Isoflurane anesthesia was used for supine fixation. Briefly, transthoracic two-dimensional motion mode-echocardiography was performed using the Vevo 2100 ultrasound imaging system. LV end-diastolic interior dimension (LVID; d), end-systolic interior dimension (LVID; s), LV end-diastolic volume (LVV; d), LV end-systolic volume (LVV; s), LV end-diastolic posterior wall (LVPW; d) and LV end-systolic posterior wall (LVPW; s) were measured and ejection fraction (EF) and fractional shortening (FS) values were calculated using the Vevo 2100 program.

### Reverse transcriptase PCR and quantitative real-time PCR

Total RNA was extracted from mice brain tissue using Trizol reagent (Invitrogen) according to the manufacturer's protocol. Complementary DNA (cDNA) was synthesized from total RNA (1 μg) using a RevertAid First Strand cDNA Synthesis Kit (Thermo Fisher Scientific, K1622) and oligo dT primers. Quantitative PCR was carried out on a Quant Studio 3 system (Thermo Fisher Scientific) using Power SYBR Green PCR Master Mix (4,367,659, Thermo Fisher Scientific) and analyzed with the comparative cycle threshold method. Primers for each gene are as follows: *COX4* primers: Forward: 5′-CCT TCT GCA CAG AAC TCA ACG C-3′ and Reverse: 5′-AGG TCT CAT TGA ACT GGA GCC G-3′; *CYC1* primers: Forward: 5′-CAG CTT CCA TTG CGG ACA C-3′ and Reverse: 5′-GGC ACT CAC GGC AGA ATG AA-3′; *Ndufs2* primers: Forward: 5′-TTT CGG GAG CTG TCA TGT ACC-3′ and Reverse: 5′-TGG TCA CCG CTT TTT CCT TCA-3′; *COX5a* primers: Forward: 5′-GCC GCT GTC TGT TCC ATT C-3′ and Reverse: 5′-CCA GGC ATC AAT GTC TGG CT-3′.

### RNA-seq and analysis

Heart tissues from EIIa-tg, Myh6-tg mice, and age-matched control mice were used for RNA extraction. An amount of 1 μg total RNA per sample was processed for library preparation using NEBNext UltraTM RNA Library Prep Kit for Illumina (NEB, USA) following the manufacturer’s recommendations. Libraries were sequenced on an Illumina NovaSeq platform to generate 150 bp paired-end reads. All total reads were mapped to GRCm38 by Hisat2. Quantification of gene expression levels was estimated by fragments per kilobase of transcript per million fragments mapped. Differential expression analysis was performed using the DESeq2. The resulting *P* values were adjusted (*P*_adj) using Benjamini and Hochberg’s approach for controlling the false discovery rate. Genes with *P*_adj < 0.01 and |FoldChange|≥ 2 were assigned as significantly differentially expressed. Gene Ontology and Kyoto Encyclopedia of Genes and Genomes enrichment analysis of the differentially expressed genes (DEGs) was implemented by the r packages of clusterProfiler. Gene Set Enrichment Analysis was also performed using the r packages of clusterProfiler.

### Mitochondrial enzyme activity assay

Mitochondrial enzyme assays were performed with ventricular mitochondria isolated from fresh mice hearts according to the instructions of the kit (BC0630, Solarbio). Either intact or sonicated mitochondria were used for enzyme assays in assay buffer at 37 ℃ using a cuvette spectrophotometer in kinetic mode.

### Statistical analysis

Data were analyzed using the Prism 8 (GraphPad) software. Two-tailed Student’s t-test was used to compare two groups and one-way ANOVA followed with Tukey’s multiple comparisons test was performed for comparison of more than two groups. All quantification data were presented as mean ± SEM. A *P* value of less than 0.05 was considered statistically significant.

### Supplementary Information

**Additional file 1: Video S1.** Increased chest movements and rapid breathing in the later stages of disease progression in the EIIa-tg mice.

**Additional file 2: Table S1.** The DEGs identified in the EIIa-tg mice and Myh6-tg mice.

**Additional file 3: Fig S1.** Gene Ontology and Kyoto Encyclopedia of Genes and Genomes pathway enrichment analyses on the differentially expressed genes identified in the heart tissues from mice. Gene Ontology (GO) and Kyoto Encyclopedia of Genes and Genomes (KEGG) pathway enrichment analyses on (**A**) total differentially expressed genes (DEGs) identified in EIIa;NOTCH2NLC-(GGC)_98_ (EIIa-tg for short) mice, (**B**) total DEGs identified in Myh6;NOTCH2NLC-(GGC)_98_ (Myh6-tg for short) mice, (**C**) DEGs exclusively identified in EIIa-tg mice, (**D)** DEGs shared in both EIIa-tg mice and Myh6-tg mice, and (**E**) DEGs exclusively identified in Myh6-tg mice. **Fig S2.** The full western blot images for figures 1B, 3B, C and 6B. **Table S2. **Detailed echocardiography data of NIID patients.

## Data Availability

All data and materials are available on reasonable request. The transcriptome sequencing data are available in Gene Expression Omnibus (GEO) database (GSE241464). DEGs lists are provided in the Additional file 2: Table S1. The full western blots are supplied in the Additional file 3: Fig S2.

## Abbreviations

NIID: Neuronal intranuclear inclusion disease
UTR: Untranslated region
PolyG: NOTCH2NLC-polyGlycine
OPDM 3: Oculopharyngodistal myopathy type 3
*NOTCH2NLC-(GGC)*_*98*_: *NOTCH2NLC* with 98 GGC repeats
EIIa-tg: EIIa;*NOTCH2NLC-(GGC)*_*98*_ transgenic
Myh6-tg: Myh6;*NOTCH2NLC-(GGC)*_*98*_ transgenic
P30: Postnatal days 30
P60: Postnatal days 60
P90: Postnatal days 90
LV: Left ventricular
LVID; d: Internal dimension at end-diastole
LVID; s: Internal dimension at end-systole
LVV; d: LV volume at end-diastole
LVV; s: LV volume at end-systole
EF: LV ejection fraction
FS: LV fractional shortening
LVPW; D: LV posterior wall thickness at end-diastole
LVPW; S: LV posterior wall thickness at end-systole
DEGs: Differentially expressed genes
GO: Gene ontology
KEGG: Kyoto Encyclopedia of Genes and Genomes
GSEA: Gene Set Enrichment Analysis
qPCR: Quantitative PCR
