# Supplementary material for: Expression of expanded GGC repeats within NOTCH2NLC causes cardiac dysfunction in mouse models
Source: Cell Biosci. 2023 Aug 29;13:157. doi: 10.1186/s13578-023-01111-6 (PMC10466825; doi:10.1186/s13578-023-01111-6)
Supplement: Supplementary file 3 — Additional file 3: Fig S1. Gene Ontology and Kyoto Encyclopedia of Genes and Genomes pathway enrichment analyses on the differentially expressed genes identified in the heart tissues from mice. Gene Ontology (GO) and Kyoto Encyclopedia of Genes and Genomes (KEGG) pathway enrichment analyses on (A) total differentially expressed genes (DEGs) identified in EIIa;NOTCH2NLC-(GGC)98 (EIIa-tg for short) mice, (B) total DEGs identified in Myh6;NOTCH2NLC-(GGC)98 (Myh6-tg for short) mice, (C) DEGs exclusively identified in EIIa-tg mice, (D) DEGs shared in both EIIa-tg mice and Myh6-tg mice, and (E) DEGs exclusively identified in Myh6-tg mice. Fig S2. The full western blot images for figures 1B, 3B, C and 6B. Table S2. Detailed echocardiography data of NIID patients. [file 13578_2023_1111_MOESM3_ESM.docx]

**Additional files for**

**Expression of expanded GGC repeats within *NOTCH2NLC* causes cardiac dysfunction in mouse model**

Yongcheng Pan^1,2^, Ying Jiang^3^, Juan Wan^4^, Zhengmao Hu^3^, Hong Jiang^1,2,5^, Lu Shen^1,2,5^, Beisha Tang^1,2,4,5^, Yun Tian^6*^, Qiong Liu^1,2*^

Correspondence to: Qiong Liu (Lqiong66@csu.edu.cn) or Yun Tian (tianyun294@126.com)

Fig S1
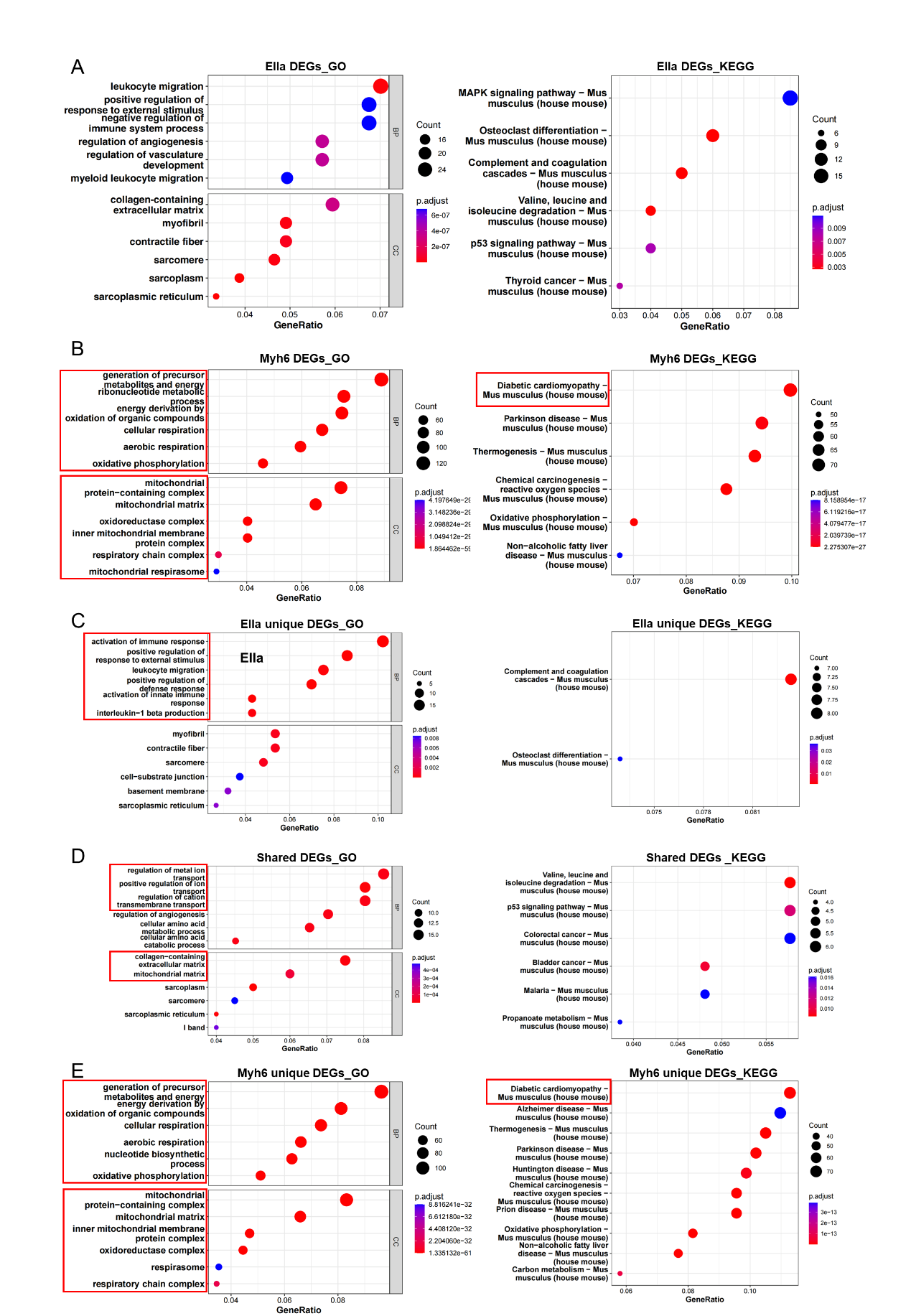


**Fig S1.** Gene Ontology and Kyoto Encyclopedia of Genes and Genomes pathway enrichment analyses on the differentially expressed genes identified in the heart tissues from mice.

Gene Ontology (GO) and Kyoto Encyclopedia of Genes and Genomes (KEGG) pathway enrichment analyses on (**A**) total differentially expressed genes (DEGs) identified in EIIa;NOTCH2NLC-(GGC)_98_ (EIIa-tg for short) mice, (**B**) total DEGs identified in Myh6;NOTCH2NLC-(GGC)_98_ (Myh6-tg for short) mice, (**C**) DEGs exclusively identified in EIIa-tg mice, (**D)** DEGs shared in both EIIa-tg mice and Myh6-tg mice, and (**E**) DEGs exclusively identified in Myh6-tg mice.

**Table S2.** Detailed echocardiography data of NIID patients.

| Case No. | Sex | Age | Disease duration | HR, beats/min | LA, mm | LV, mm | IVS, mm | EF,  % | Wall motion |
| --- | --- | --- | --- | --- | --- | --- | --- | --- | --- |
| 1 | M | 66 | 30 | 103 | 23 | 43 | 8 | 69 | normal |
| 2 | M | 60 | 3 | 76 | 33 | 41 | 13 | 63 | normal |
| 3 | F | 62 | 10 | 105 | 29 | 40 | 8 | 75 | normal |
| 4 | M | 42 | 13 | 68 | 38 | 51 | 9 | 63 | normal |
| 5 | M | 56 | 1 | 65 | 41 | 49 | 12 | 70 | normal |
| 6 | M | 61 | 11 | 54 | 29 | 46 | 9 | 64 | normal |
| 7 | F | 65 | 39 | 74 | 24 | 40 | 8 | 63 | normal |

EF=ejection fraction; HR=heat rate; IVS=interventricular septum; NIID= neuronal intranucler inclusion disease; LA=left atrium; LV=left ventricle.

**Fig S2**


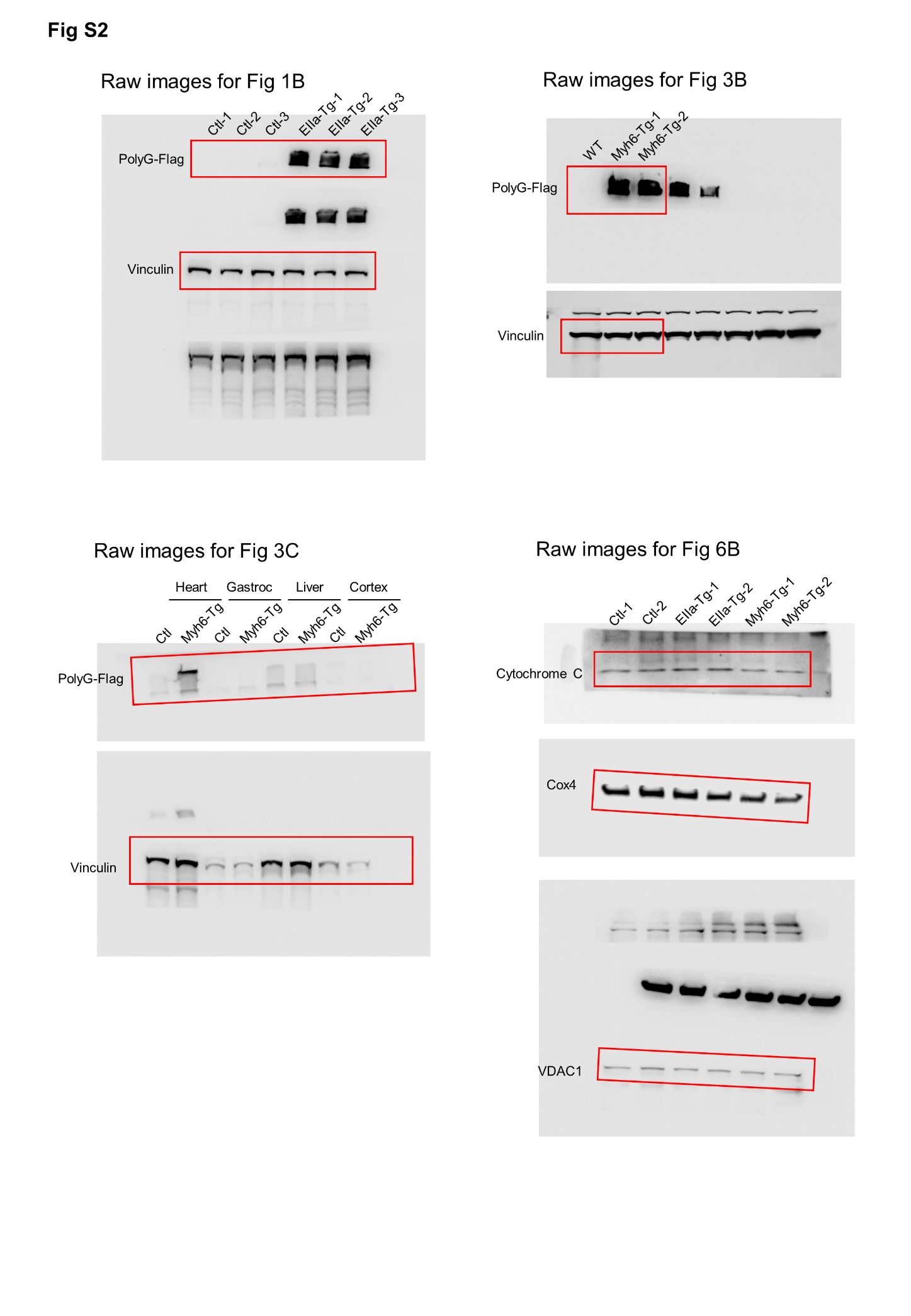


**Fig S2.** The full western blot images for figure 1B, 3B, 3C and 6B.
